# Comparison of Xylazine and Lidocaine Infusion versus Medetomidine Continuous Rate Infusion during General Anesthesia with Isoflurane in Horses Undergoing Emergency Laparotomy

**DOI:** 10.3390/vetsci11050196

**Published:** 2024-04-29

**Authors:** Paola Straticò, Giulia Guerri, Lorenza Bandera, Gianluca Celani, Laura Di Nunzio, Lucio Petrizzi, Vincenzo Varasano

**Affiliations:** Department of Veterinary Medicine, University of Teramo, 64100 Teramo, Italy; pstratico@unite.it (P.S.); gguerri@unite.it (G.G.); gcelani@unite.it (G.C.); ldinunzio@unite.it (L.D.N.); lpetrizzi@unite.it (L.P.); vvarasano@unite.it (V.V.)

**Keywords:** general anesthesia, laparotomy, α2-agonists, lidocaine, horse

## Abstract

**Simple Summary:**

General anesthesia has several main goals, including pain management and cardiovascular support. In this retrospective study, we compared two anesthetic protocols for general anesthesia with isoflurane in horses undergoing emergency laparotomy. In the first, xylazine was administered followed by an intraoperative infusion of lidocaine. In the latter, medetomidine was used for preoperative sedation and intraoperative infusion. We reviewed medical records and registered intraoperative variables, recovery time and quality, and short-term outcomes. Horses receiving preanesthetic xylazine sedation followed by intraoperative infusion showed more respiratory depression and a higher heart rate at the beginning of the surgery prior to lidocaine infusion and at the end of the surgery after the discontinuation of lidocaine infusion, which may indicate insufficient analgesia during this time. Arterial pressure was better maintained when medetomidine was used. Recovery quality was similar, with a longer time required for horses receiving medetomidine. We can conclude that both protocols are suitable for general anesthesia with isoflurane in horses undergoing emergency laparotomy. Medetomidine provided more efficient analgesia at the beginning and end of surgery with longer recovery times, more suitable for anxious and young horses to prevent self-injury during this phase.

**Abstract:**

(1) The main goals of general anesthesia include pain management and a safe anesthetic protocol for smooth recovery. In this retrospective study, we compared two anesthetic protocols for general anesthesia with isoflurane during emergency laparotomy: sedation with xylazine and the intraoperative infusion of lidocaine (X group) versus medetomidine as a preoperative sedation and intraoperative infusion (M group). (2) The medical records of horses who underwent emergency laparotomies between 2016 and 2023 were reviewed. According to the anesthetic protocol, patients were allocated to the X or M groups. Data about the horse, signalment, history, and anesthetic variables were analyzed. (3) Group X had a significantly higher heart rate (HR), lower respiratory rate (RR) and mean and diastolic arterial pressure (MAP/DAP). A progressive increase in HR and RR was observed in both groups. Group X underwent a decrease in RR and an increase in DAP. In Group M, a decrease in MAP and DAP was observed. Group M exhibited a longer recovery time with similar recovery scores. Both protocols provided safe anesthesia for emergency laparotomy, with minor cardiovascular and respiratory depression. Minor respiratory depression was detected when xylazine was used, while recovery was longer with medetomidine.

## 1. Introduction

General anesthesia during an emergency laparotomy in equine medicine is still of great concern, despite improvements to the quality of induction and recovery, monitoring details during the procedure, and pain management during the perioperative period in recent decades. The mortality rate has decreased consistently, according to recent reports, from 35.3% [1] to 31.4% [2] and 7.8% [3]. Inconsistencies between studies are mainly due to differences in data recording, inclusion/exclusion criteria, primary surgical lesion, and patient condition, as well as varying definitions of the perioperative period [4]. Some authors assert that about 15% of all fatalities are directly related to anesthesia [2], significantly less than previous reports [5]. 

Anesthetic protocols aim to reach a stable plan with the least cardiovascular and respiratory depressions. Although there is no correct protocol for general anesthesia, in equine medicine, combining an α2 agonist and opioid with a volatile agent is the best option for obtaining an optimal balance between drugs and reducing their adverse effects.

Xylazine is the least selective among α2-agonists [6], providing good but short-lasting sedation and analgesia. It is widely used in premedication [7,8] with a sparing effect on isoflurane or halothane, if used as a bolus [9,10], or in continuous rate infusion (CRI) during partial intravenous anesthesia (PIVA) with minimal cardiopulmonary effects [11].

Medetomidine is more selective than xylazine [6]. Although not approved for equids, its pharmacokinetics and pharmacodynamic properties make it suitable for PIVA protocols during routine procedures [12,13]. In experimental studies, medetomidine has demonstrated good sparing effects on isoflurane- and desflurane-anesthetized ponies [12] and horses [14] with good recovery scores [15,16,17,18].

Lidocaine is a local anesthetic agent that reduces the need for inhalation agents in animals [19,20,21,22]. It reduces the minimum alveolar concentration (MAC) of isoflurane when administered as a loading dose (2.5 mg/kg i.v. for 15 min) followed by a CRI (0.05 mg/kg/min i.v.) [21]. Concerns about the intraoperative CRI use of lidocaine in horses are mainly related to possible complications during recovery, as it may increase ataxia [23]. Due to its analgesic and anti-inflammatory properties when absorbed systemically [24,25,26] and its potential prokinetic activity on the gastroenteric tract [27,28,29,30], its use is recommended during an emergency laparotomy.

In this study, we compared two commonly used anesthetic protocols for emergency laparotomy based on their depth of anesthesia and recovery characteristics.

## 2. Materials and Methods

Anesthetic records of horses referred to the Veterinary Teaching Hospital of the University of Teramo from 2016 to 2023 were retrospectively reviewed. 

Adult horses (>1 year old) referred for colic syndrome and undergoing exploratory laparotomy were included in the study. Horses were assigned to the X or M groups based on the anesthetic protocol used:

- The X group received preoperative sedation with xylazine (0.6 mg/kg i.v.) followed by butorphanol (0.02 mg/kg i.v.) and an intraoperative CRI of lidocaine (0.05 mg/kg/min i.v.) [31];
- The M group received medetomidine (7 μg/kg i.v.) followed by morphine (0.1 mg/kg i.m.) and an intraoperative CRI of medetomidine (3.5 μg/kg/h i.v.) [13].

In each group, general anesthesia induction was performed with an intravenous bolus of ketamine (2.2 mg/kg) and midazolam (0.06 mg/kg). Once recumbent, an endotracheal tube was inserted, and the horse was hoisted onto a surgical table in dorsal recumbency. The horse was then moved to the surgery room and connected to a large animal anesthetic circuit (Surgivet DHV 1000, Medical Device Depot, Fairview, PA). General anesthesia was obtained with isoflurane in a mixture of O_2_ and air (2:1). The above-mentioned CRI (either lidocaine or medetomidine) was associated with inhalation anesthesia. The reservoir bag was either 15 L or 30 L according to the horse’s tidal volume and was filled with isoflurane, air, and O_2_. The vaporizer setting was set at 3% until a MAC of ISO of 1.1 was reached, then adjusted to reflect the clinical parameters. A crystalloid infusion was provided for the entire duration of anesthesia, starting with a rate of 5 mL/kg/h and adjusted according to the preoperative packed cell volume (PCV), urine output, and mean arterial blood pressure. Lidocaine and medetomidine infusion were started immediately, always within 15 min of anesthesia induction.

Intraoperative anesthetic monitoring was obtained with a multiparametric monitor (GE Healthcare B 650, GE Health Care, Treviglio (MI), Italy) and manually recorded every 5 min.

An arterial catheter of 22 G was placed in the facial artery to monitor invasive arterial pressure. Dobutamine infusion was initiated as soon as the patients were connected to the anesthetic circuit with an initial CRI rate of 0.5 μg/kg/min, which was adjusted as needed [31].

If the horse showed nystagmus, a bolus of ketamine was administered (0.2–0.5 mg/kg i.v.). During excitation (spontaneous ear movement, swallowing, or other spontaneous movements) a bolus of thiopental was administered (0.5–1 mg/kg i.v.). In both cases, the anesthesia plan was considered too light and isoflurane vaporization was increased to obtain an FE ISO increase of 0.1%.

Twenty to thirty minutes before the end of the surgery, lidocaine infusion was stopped [22]. Medetomidine infusion was discontinued together with the interruption of inhalant anesthesia. 

At the end of the surgery, the horses were moved to the recovery room and received a bolus of either xylazine (0.2 mg/kg i.v.) or medetomidine (2 μg/kg i.v.). O_2_ flow-by (15 L/min) was administered through the endotracheal tube until its removal once the swallowing reflex was restored. In all cases, recovery was unassisted and scored on a scale ranging from 1 (excellent recovery) to 5 (very poor recovery) (Table 1) [32].

For each horse, we recorded every 5 minutes the sex, age (years), weight (kg), duration of anesthesia and surgery (minutes), time to extubation (minutes), time to sternal recumbency (minutes), time to standing (minutes), recovery score, heart and respiratory rate (HR-RR), systolic, diastolic and mean invasive arterial pressure (SAP, DAP, MAP) (mmHg), the minimum alveolar concentration of isoflurane (MAC-Iso), and End-Expiratory Carbon Dioxide Tension (PET-CO2), and the type and number of intraoperative top-ups. RR was calculated from the capnogram. We obtained MAC-Iso and PET-CO2 through a side-stream gas sampler that was connected to the Y-piece bifurcation of the rebreathing system.

Short-term outcomes were also considered: positive (the horse recovered from anesthesia and was discharged without major complications), euthanasia (the horse underwent euthanasia either intra- or postoperatively), or death (the horse recovered from anesthesia but died spontaneously).

Data were tested for normality using a Shapiro–Wilk test. For statistical analysis, an unpaired T test or Mann–Whitney U test was used to compare the continuous variables between Groups X and M. A repeated measures ANOVA or Friedman test was used to highlight changes over time from T0 (connection to the anesthetic machine) to T90. For categorical variables, a χ^2^ test was used. The significance level was set to *p* < 0.05. 

## 3. Results

We determined that 91 medical records were eligible, with 45 belonging to Group X and 46 to Group M.

No differences were detected between the groups for age, weight, or sex (Table 2).

**Figure 1:**
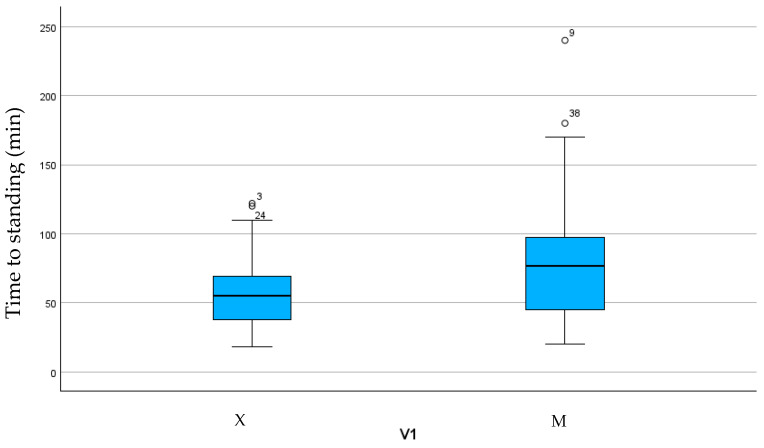
Box plot showing time to standing in the two groups.

The duration of anesthesia and surgery, time to extubation, and time spent in sternal recumbency were not statistically different between the groups (Table 3). Group M showed a longer time to standing (*p* < 0.05) (Figure 1). 

Despite the longer time to standing, the recovery score was similar between the groups, with most frequencies expressed for scores 1 and 2 (Table 4). Information was not retrievable from clinical records for 15 out of 45 cases in Group X and 25 out of 46 cases in Group M.

When short-term outcomes were considered, both groups showed similar results (Table 5). In two cases, the required information was not retrievable from the available clinical records.

**Figure 2:**
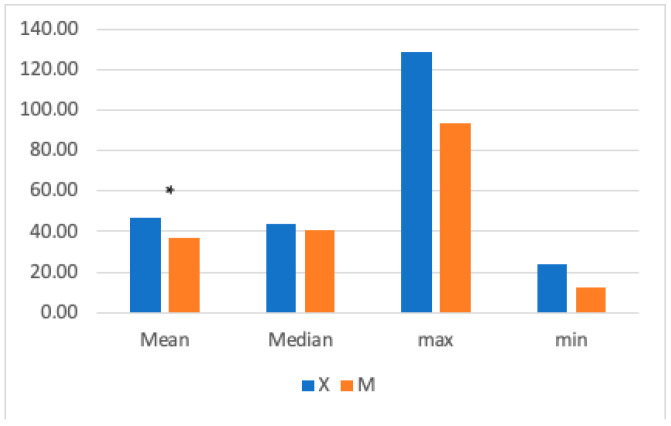
The graph shows the mean, median, maximum, and minimum heart rate (HR) values in Groups X and M. The vertical axis refers to beats/min. * represents that the difference in mean was statistically significant (independent sample *t*-test, significance set to *p* < 0.05).

The mean HR was significantly higher in Group X (47.54 ± 13.25 bpm) compared to GroupM (37 ± 12.83 bpm) (Figure 2). Significant differences were found between 10- and 35-min post-anesthetic induction, with higher HR values in Group X.

**Figure 3:**
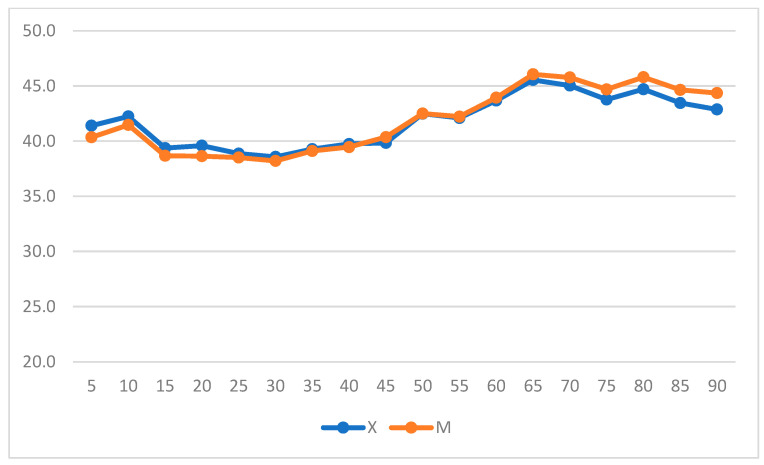
Heart rate (HR) variation over time in Groups X and M. The vertical axis refers to beats/min, and the horizontal axis refers to time expressed in minutes.

When intragroup variation was considered, both groups experienced significant increases in HR (*p* = 0.019 in Group X; *p* = 0.011 in Group M) (Figure 3) (repeated measures ANOVA, with statistical significance was set to *p* < 0.05).

**Figure 4:**
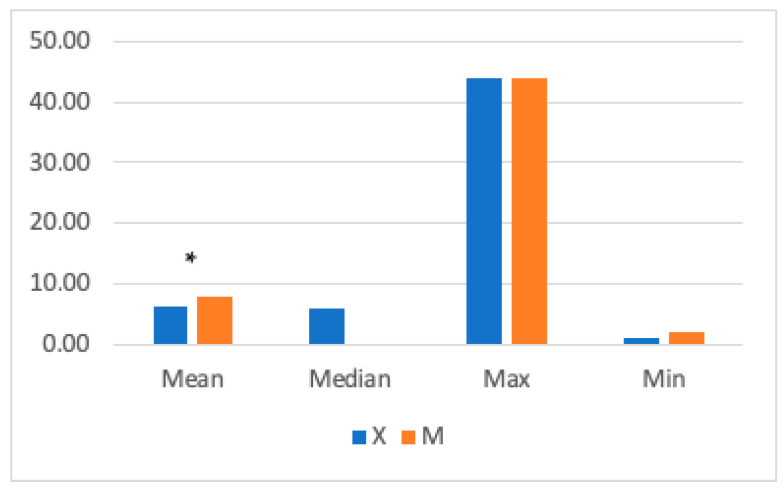
The graph shows mean, median, and maximum and minimum respiratory rate (RR) values in Groups X and M. The vertical axis refers to breaths/min. * represents that differences in mean were statistically significant (independent sample *t*-test, significance level set to *p* < 0.05).

The mean RR was significantly higher in Group M (7± 4.9 breaths/min) compared to Group X (6.39 ± 3.18 breaths/min) (Figure 4). Significant differences were found between 15- and 30-min post-anesthetic induction, with higher RR values in Group M.

**Figure 5:**
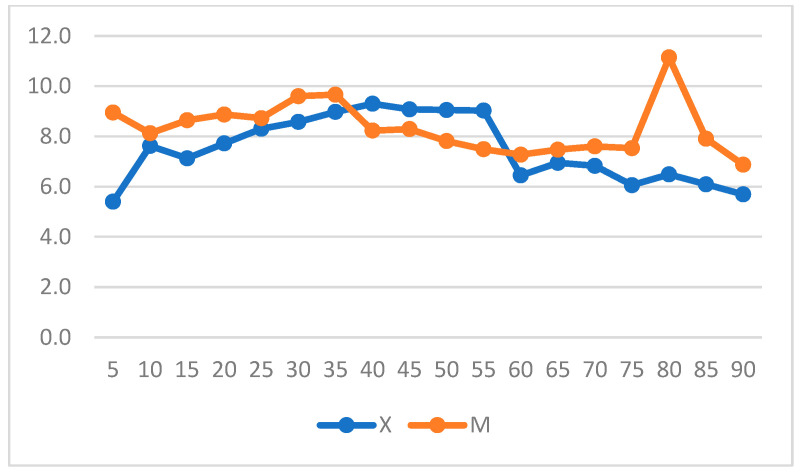
Respiratory rate (RR) variation over time in Groups X and M. The vertical axis refers to respiratory acts/min and the horizontal axis refers to time expressed in minutes.

When intragroup variation was considered, a significant increase in RR from T0 to T55 was observed over time in Group X (Figure 5) (*p* = 0.001). 

**Figure 6:**
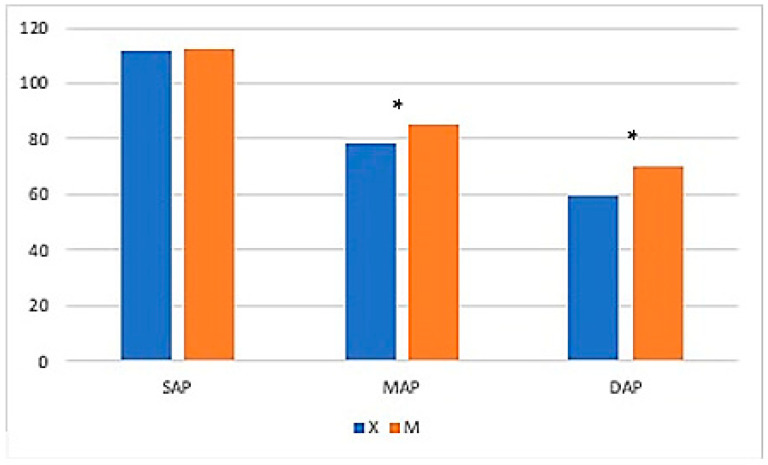
The graph shows mean systolic arterial pressure (SAP), mean arterial pressure (MAP), and diastolic arterial pressure (DAP) values in Groups X and M. The vertical axis refers to mmHg. * represents that differences in mean were statistically significant (independent sample *t*-test, significance level set to *p* < 0.05).

No differences were found for MAC-Iso, PET-CO_2_, or SAP (*p* > 0.05) (Table 6). Overall, mean MAP values were significantly higher in Group M and at time points from 15 to 45 min. Mean DAP values were higher in Group M and at the following time points: T20—25–45–50–90 min (Figure 6).

**Figure 7:**
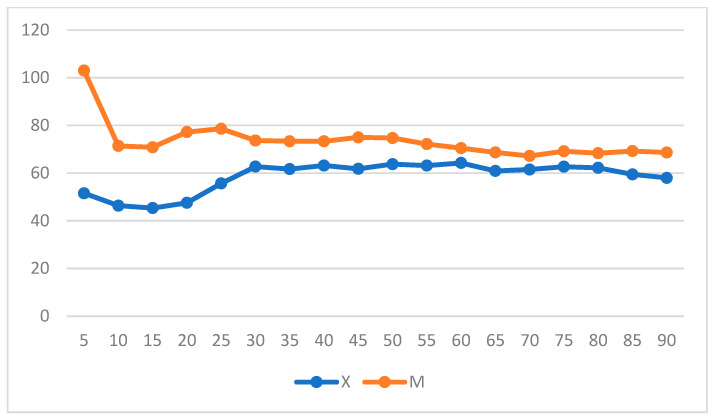
Diastolic arterial pressure (DAP) variation over time in Groups X and M. The vertical axis refers to mmHg. The horizontal axis refers to time expressed in minutes.

**Figure 8:**
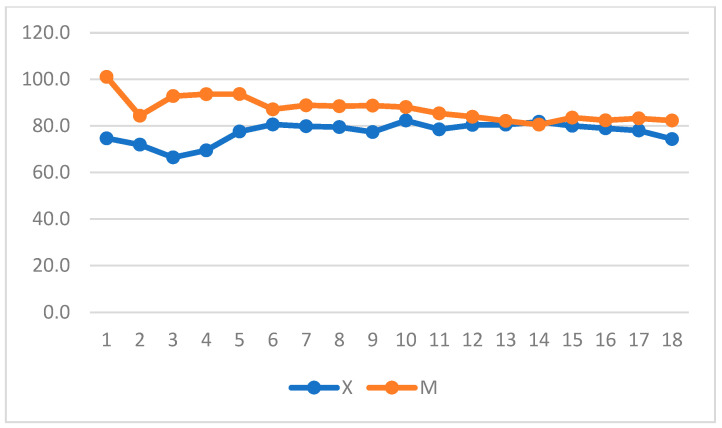
Mean arterial pressure (MAP) variation over time in Groups X and M. The vertical axis refers to mmHg. The horizontal axis refers to time expressed in minutes.

When intragroup variation was considered in Group X, a significant increase in DAP was observed, whereas a decrease in DAP and MAP was observed in Group M (*p* = 0.004 and *p* = 0.05) and over time in Group X (Figure 7 and Figure 8) (repeated measures ANOVA, with statistical significance set to *p* < 0.05). 

The number of top-ups was similar between groups, with a higher amount of ketamine administered to Group M than thiopental to Group X (Group X: thiopental = 2 horses; ketamine = 20 horses; thiopental + ketamine = 5; Group M: thiopental = 4 horses; ketamine = 15 horses; thiopental + ketamine = 5).

## 4. Discussion

In this retrospective study, two anesthetic protocols were compared for general anesthesia in horses undergoing emergency exploratory laparotomy. The first protocol (Group X) was based on preoperative sedation with xylazine (0.6 mg/kg i.v.) followed by butorphanol (0.02 mg/kg i.v.) and an intraoperative CRI of lidocaine (0.05 mg/kg/min i.v.). The second protocol (Group M) was based on preoperative sedation with medetomidine (7 μg/kg i.v.) followed by morphine (0.1 mg/kg i.m.) and an intraoperative CRI of medetomidine (3.5 μg/kg/h i.v.). A significant difference was found for mean HR, with lower values in Group M, and RR, with lower values in Group X. Furthermore, MAP and DAP values were lower in Group X. Despite similar recovery quality, Group X showed the quickest and worst recoveries (scores 4 and 5), which were not observed in Group M.

Even though there is no perfect protocol, general anesthesia during emergency laparotomy for colic syndrome intervention aims to maintain or re-establish cardiovascular function and manage pain. 

The use of α_2_-agonists during general anesthesia in equids is known to have a sparing effect on isoflurane and reduce respiratory depression caused by inhalation anesthesia. 

Xylazine and medetomidine have different sedative powers in equids because of their different selectivity. However, both are used as sedatives for single bolus administration or for PIVA in horses [13,33,34,35,36]. Their short half-lives make them particularly suitable for continuous rate infusion, with reduced adverse effects compared to single bolus administration [37,38,39].

Butorphanol and morphine are associated with the bolus administration of either xylazine or medetomidine before surgery to improve α_2_-agonists’ effects without affecting the MAC of inhalation anesthetics [40,41,42,43].

A previous study [44] found no major differences between intraoperative lidocaine bolus administration followed by CRI or CRI alone. However, we decided not to use a loading dose of lidocaine because it is considered safer to avoid high drug plasma concentrations, especially in long surgeries with compromised horses whose conditions may alter lidocaine pharmacokinetics. Despite lidocaine’s advantages during anesthesia, the drug’s toxic range is highly variable. Clinical signs of intoxication may appear when plasma levels are high [45]. Moreover, it is unclear whether gastrointestinal injuries affect drug metabolism and facilitate drug accumulation, especially if renal and hepatic function are compromised [29,46,47]. Individual differences in cardiac output and sympathetic tone are responsible for high individual variability in plasma drug concentrations among horses receiving lidocaine. Reduced cardiac output, which frequently occurs in colic horses, could be the reason for lower hepatic and renal metabolic function and higher plasma drug concentrations. On the other hand, horses suffering from pain have increased sympathetic tone, higher cardiac output, and faster drug metabolism [29].

We observed a reduction in mean HR in Group M, and this difference was more evident from 10 to 35 min after anesthesia induction. We consider this a result of a stronger analgesia in Group M. These results are in agreement with those observed by Nannarone and Spadavecchia in 2015 [44]. They determined that horses that did not receive a lidocaine bolus had a higher HR mean in the first 30 min of anesthesia compared to horses that received a loading dose of lidocaine, probably due to lower plasma lidocaine levels and lower analgesia. Additionally, Ringer et al. [32] demonstrated a lower HR mean value in horses that received an intraoperative CRI of medetomidine compared to those receiving a CRI of lidocaine. Despite HR being below normal limits at some time points, there was no need for correction since systemic blood pressure was within normal limits. 

Group M tended to have better systemic blood pressure with higher mean values, particularly at the beginning of anesthesia. These data agree with a previous study [32] comparing an anesthetic protocol with lidocaine as a loading dose followed by CRI to preoperative sedation and intraoperative CRI with medetomidine for general anesthesia during elective surgeries. Dobutamine infusion was administered in all cases [31]. The same results were obtained by Creighton et al. [16] when medetomidine CRI was compared to a xylazine bolus in a randomized controlled trial. These measures provide important advantages and protective factors against hypotension [48,49,50], which contributes to post-anesthetic morbidity and death. Despite clinical evidence, care must be exercised when judging these data, as information about the use of colloids (type and dosage) is often lacking in the clinical records and for this reason not analyzed.

MAC-Iso was evaluated in previous studies comparing different anesthetic protocols [21,44]. Nannarone and Spadavecchia [31] investigated the effects of xylazine administered as a preoperative bolus followed by xylazine administered as an intraoperative CRI in horses undergoing emergency laparotomy with isoflurane. They did not detect any differences in MAC-Iso between groups despite observing a lower sparing effect on isoflurane than Dzikiti [21], who tested a higher loading dose of xylazine in anesthetized healthy horses. In this retrospective study, MAC ISO did not differ between groups; therefore, we cannot conclude that either protocol has a more pronounced sparing effect on isoflurane consumption. On the other hand, we observed a lower RR in Group X. This finding can be ascribed to more profound anesthetic plans or pronounced respiratory depression. Unfortunately, blood gas analysis, cardiac output, and tissue O_2_ delivery information was unavailable. For this reason, we cannot ascribe beneficial effects to the reduced mean HR value in Group M since PaO_2_ and PaCO_2_ values were unavailable. Although the respiratory response to α2-agonists is highly variable in anesthetized horses, our results were similar to those reported by Creighton et al. despite similar isoflurane concentrations between groups [16].

The total number of top-ups was similar between groups, with Group M receiving more ketamine bolus and Group X more thiopental bolus. When we analyzed the timing of administration of top-ups, we noticed that they were administered more frequently in Group X within 15 min of induction or after lidocaine infusion interruption. In Group M, we could not identify a similar distribution of the events. The different timings, rather than the number of top-ups, can be explained by the less analgesic properties with the X protocol at the beginning of the intervention, probably before an effective lidocaine plasma level was reached, and after its interruption 20–30 min before the end of surgery [22], when it decreases rapidly due to lidocaine’s short half-life [51,52].

The use of two different opioids instead of one is a limitation of the study because another variable is introduced. Both butorphanol and morphine are reported to have a short duration when used together with α2-agonists or alone. Morphine has a longer duration of action, so it may have influenced the number of top ups during the second half of the anesthesia. Since it takes 30 min to reach effective plasma levels and it was administered intramuscularly after sedation, its analgesic effects would not be a reason for less top-ups during the first part of the procedure.

We observed a significantly longer time to stand and recovery phase in Group M than in Group X despite the recovery score not being statistically different between groups. Most recoveries were scored as excellent or good. Although we did not observe any catastrophic injury during this phase, the worst recoveries were encountered in Group X. We must highlight that all horses in Group X received a bolus of xylazine once in the recovery room. Previous studies also emphasize the need for this measure [53,54] to improve recovery scores from general anesthesia during elective surgery. In all cases, recoveries were unassisted since no consensus was found in the literature concerning this aspect [55,56,57]. Shorter recoveries without differences in quality were observed when xylazine was administered as a bolus at the end of surgery [55,56]. This finding may suggest that medetomidine is predisposed to longer, quieter recoveries from general anesthesia for emergency laparotomy. 

Although the outcome was not statistically significant between protocols, care must be exercised when evaluating emergency laparotomy outcomes as colic syndrome is multifactorial and a variety of aspects are correlated to surgical intervention success rates [58,59,60,61,62,63,64,65,66,67,68]. In most cases, horses were discharged from hospitals without major complications. Intraoperative euthanasia was related to primary gastrointestinal disorders and no intraoperative deaths were observed. 

Besides those already mentioned, this study has several limitations. 

Given its retrospective nature, some data may be missing, and recording strategies can vary across clinical practices. No blindness or randomization was achieved, and assignment to either group depended on the anesthetists’ preferences. Furthermore, we did not perform a power analysis to enhance the results. Although the sample size appears to be low, this is in line with previous retrospective studies on the topic [69]. The primary abdominal disorder requiring surgery may affect the results because it affects common intraoperative variables associated with cardiovascular and electrolyte imbalances.

## 5. Conclusions

According to the results obtained from this retrospective study, we can conclude that both protocols provide stable anesthesia with a good cardiovascular balance and minor respiratory depression. The intraoperative CRI of medetomidine produces more pronounced analgesia, especially at the beginning and end of surgery. Good recovery quality was obtained from both protocols. When medetomidine infusion was used, longer standing times during the recovery phase were required. Since individual temperament affects recovery scores and a longer recovery duration is associated with better recovery [70], medetomidine is preferred to xylazine in anxious or young horses to allow more time for recovery from general anesthesia. Moreover, the registration status of the animal (either food-producing or non-food-producing) must be considered according to the country’s legislation. For a better understanding on this topic and objective results, prospective studies should standardize primary lesions requiring surgery and randomize anesthetic protocols.

## Figures and Tables

**Table 1:** Quality of recovery from anesthesia [32].

Score		
1	Excellent recovery	The horse is capable of standing at first attempt.
2	Very good	The horse remained calm; two attempts to stand.
3	Good	The horse remained calm; >2 attempts.
4	Poor	Excitement during recovery with danger to the horse; >2 attempts.
5	Very poor	Severe excitement during recovery with injury to the horse.

**Table 2:** Comparison of age, weight, and sex distribution between Groups X and M ^1^.

Variable	Group X	Group M	*p*-Value
Age (years)	14 ± 10 years	10 ± 5	0.270
Weight	460.3 ± 136.53	462.5 ± 115	0.215
Female	39.1% (18)	30.4% (14)	0.25
Male	60.9% (27)	43.5% (20)	
Unknown gender	0	52.2% (12)	

^1^ Values are expressed as mean ± standard deviation for age and weight, and as percentage (absolute values) for female, male, and unknown sex. For continuous numerical variables (age and weight) an independent sample *t*-test was used. For categorical variables (female, male, and unknown gender), an χ^2^ test was used. Statistical significance was set to *p* < 0.05.

**Table 3:** Duration of anesthesia, duration of surgery, time to extubation, and time spent in sternal recumbency in Groups X and M ^1^.

Outcome	Group X % (*n*)	Group M % (*n*)	*p*-Value
Duration of anesthesia (min)	105.4 ± 36.58	111.1 ± 38.42	0.664
Duration of surgery (min)	79.6 ± 36.43	90.1 ± 32.70	0.598
Time to extubation (min)	18.8 ± 13.87	16.8 ± 10.6	0.055
Time in sternal recumbency (min)	35.6 ± 17.23	43.42 ± 17.04	0.815
Time to standing	58.6 ± 27.82	79.5 ± 49.18	0.042 *

^1^ Values are expressed as mean ± standard deviation. An independent sample *t*-test was used. * Statistical significance was set to *p* < 0.05.

**Table 4:** Frequency of occurrence of recovery score between groups ^1^.

Score	Group X % (*n*)	Group M % (*n*)	*p*-Value
1	41.9% (13)	90.5% (19)	0.24
2	19.4% (6)	4.8% (1)	
3	16.1% (5)	4.8% (1)	
4	16.1% (5)	0	
5	6.4% (2)	0	

^1^ Values are expressed as percentages (absolute values). An χ^2^ test was used. Statistical significance was set to *p* < 0.05.

**Table 5:** Short-term outcome comparison between Groups X and M ^1^.

Outcome	Group X % (*n*) (*n* = 5)	Group M % (*n*) (*n* = 46)	*p*-Value
Positive	66.7% (30)	71.7% (33)	0.255
Intraoperative euthanasia	33.3% (15)	21.7% (10)	
Post-operative death	0	6.6% (3)	

^1^ Values are expressed as percentages (absolute values). An χ^2^ test was used. Statistical significance was set to *p* < 0.05.

**Table 6:** Comparison between Groups X and M for MAC-Iso, PET-CO_2_, SAP, MAP, and DAP. * represents that difference in mean was statistically significant.

Variable	Group X	Group M	*p*-Value
MAC-Iso	1.2 ± 0.24	1.2 ± 0.32	0.5
PET-CO_2_	42.7 ± 11.67	42 ± 8.66	0.3
SAP	111.5 ± 23	112.2 ± 25.35	0.6
MAP	78.8 ± 18.71	85 ± 21.29	0.01 *
DAP	59.68 ± 19.12	70.43 ± 20.09	0.02 *

## Data Availability

Policy restrictions relating to the owners’ and patients’ privacy affect data availability. The data presented in this study are available upon request from the corresponding author.
